# Cognitive Behavioral Therapy in Early Intervention for Psychosis: A Cost-Effectiveness Analysis

**DOI:** 10.36469/001c.162047

**Published:** 2026-06-11

**Authors:** Maximilian Duckworth, Sofia Venturini, Andres Roman-Urrestarazu

**Affiliations:** 1 Department of Psychiatry University of Cambridge, Cambridge, UK; 2 School of Health, Medicine and Life Sciences University of Hertfordshire, Hatfield, UK; 3 Department of Psychology and Cognitive Science University of Trento, Rovereto, Italy

**Keywords:** psychosis, early intervention for psychosis, cognitive behavioural therapy, health economic evaluation, cost-effectiveness analysis

## Abstract

**Background:**

Early intervention for psychosis (EIP) is a multidisciplinary service that treats individuals experiencing early psychosis. Treatment often consists of pharmacotherapy and cognitive behavioral therapy for psychosis (CBTp). While EIP demonstrates clinical efficacy and cost-effectiveness as a comprehensive package, the incremental contribution of its individual components remains unclear. With mental health services facing sustained funding pressures, understanding which EIP elements offer value for money is crucial for resource allocation decisions.

**Objectives:**

To evaluate whether CBTp used in conjunction with pharmacotherapy in EIP is cost-effective compared with pharmacotherapy alone.

**Methods:**

A cost-effectiveness analysis was conducted using a cohort-based Markov model from the societal perspective over a 3-year time horizon. Model parameters were derived from a targeted literature review. The primary outcome was the incremental cost-effectiveness ratio (ICER), expressed as the cost per quality-adjusted life-year (QALY) gained. One-way sensitivity analysis identified influential parameters and probabilistic sensitivity analysis (PSA) quantified uncertainty around the ICER.

**Results:**

CBTp as an adjunct to pharmacotherapy resulted in a gain of 0.48 QALYs in the base case compared with pharmacotherapy alone. The corresponding ICER was £32 979 per QALY gained. This currently sits above the conventional upper willingness-to-pay threshold of £30 000 per QALY. One-way sensitivity analysis identified cost parameters and utility parameters for symptom-free status as the most influential variables on the ICER. PSA showed that CBTp had approximately a 50% probability of being cost-effective at the £30 000 threshold.

**Conclusions:**

Parameter uncertainty, particularly regarding costs and health utilities, drives the marginal cost-effectiveness case for CBTp. The intervention’s proximity to conventional thresholds suggests targeted improvements in service delivery or patient selection could enhance value. CBTp could potentially be a cost-effective adjunct to pharmacotherapy in EIP; however, there is uncertainty around the results of this modeling. These findings support the targeted implementation of CBTp within EIP. Further high-quality long-term studies are recommended to strengthen the evidence base.

## BACKGROUND

Psychotic disorders are incapacitating conditions with detrimental effects on an individual’s global functioning. Psychosis is characterized by the inability to determine what is real and not real, and its symptoms can be positive (such as delusions and hallucinations) or negative (such as blunting and catatonia).^1^

First-episode psychosis (FEP) refers to when someone exhibits psychotic symptoms for the first time.^2^ Recovery trajectories vary,^3^ with some achieving complete remission while others experience chronic symptoms or episodic relapses. FEP incidence in the UK is approximately 50 per 100 000 annually, with average onset during early adulthood.^4^

Worse outcomes are associated with male sex, younger age of onset, black ethnicity, lower education, social deprivation, substance misuse, and longer duration of untreated psychosis (DUP).^5,6^ FEP significantly impacts individuals during critical life domains including education, employment, and relationships. Prolonged untreated psychosis correlates with greater brain damage,^7^ and inadequate treatment may progress to schizophrenia,^8^ with increased risks of substance misuse, homelessness, and unemployment.^9^

Direct costs to the National Health Service (NHS) for managing psychosis amount to £2 billion annually.^10^ Previous research has further estimated the annual societal cost per schizophrenia patient to reach US $30 140 in Germany and US $94 587 in Norway.^11^ High rates of physical co-morbidity contribute to life expectancy reductions of up to 20 years.^12^

Early intervention for psychosis (EIP) services provide multidisciplinary community-based treatment for patients in the early stages of a psychotic disorder.^13^ Characteristics of EIP include early detection, small patient/staff ratios, and treatment programs of up to 3 years.^14,15^ EIP delivers complex interventions, with components including pharmacotherapy, psychotherapy like cognitive behavioral therapy (CBT), family interventions, and social or vocational programs.^16^ Beyond minimizing the DUP, EIP aims to promote recovery and prevent relapses or long-term disability.^17^ Importantly, EIP services are provided by one coordinated team, instead of having patients referred to different services for each component of their treatment.^14^

EIP has been praised for being nonstigmatizing and recovery-focused.^18^ Meta-analyses demonstrate superiority over treatment as usual (TAU) for multiple outcomes.^14,17^ There is evidence that EIP as a comprehensive multidisciplinary package is cost-effective, being estimated to save the NHS £4031 per patient annually compared with TAU.^19^ A systematic review by Aceituno et al^20^ found comprehensive EIP to be cost-effective with high probability, though this evidence again pertains to integrated service packages rather than individual therapeutic components. Demonstrating that EIP is cost-effective overall does not establish the incremental value of its individual components.

The mainstay of EIP is antipsychotic medication.^21^ Second-generation antipsychotics (SGAs) are generally favored by clinical guidelines because they are associated with fewer and less intense side effects.^22^ Generally, there is a good response to antipsychotics in 40% to 50% of patients, and a partial response in 30% to 40%.^23^

National Institute for Health and Care Excellence (NICE) guidelines for EIP recommend offering patients with FEP at least 16 sessions of CBT for psychosis (CBTp) alongside antipsychotics.^24^ CBT is a broad psychological framework applied across a wide range of mental health conditions and promotes the creation of mental links between thoughts, feelings, actions, and symptoms so that individuals change how they interpret their experiences.^25^ CBTp is a specific adaptation of CBT, targeting the distress and functional impairment arising from the cognitive and perceptual disturbances in psychosis.^26^ Rather than directly challenging the content of psychotic experiences, CBTp works to develop less distressing interpretations of those experiences and reduce their impact on behavior and well-being.^26^ In the United Kingdom, CBTp is delivered in an individual format.^27^

Although EIP is the gold standard intervention for FEP in the UK,^16^ consensus on the importance of individual component contributions is lacking.^16^ While pharmacotherapy has a robust evidence base and is provided almost universally in EIP, there is conflicting evidence regarding the effectiveness of other interventions like CBTp, especially long-term,^16^ and national guidelines have thus far been based largely on expert opinion rather than robust comparison of treatment models.^16^ While cost-effectiveness evidence for EIP as a comprehensive package is growing, evidence specifically for CBTp as a discrete intervention remains limited. Previous research suggests CBTp is potentially cost-effective depending on the population and disease severity,^28,29^ though the certainty of these conclusions is limited by small effect sizes and study numbers. CBTp may be especially effective in preventing FEP in high-risk individuals and improving psychological well-being in individuals with positive symptoms.^28^

Unsurprisingly, there is a growing appreciation of the importance of maximizing health outcomes given systemic restraints, and policymakers look to economic evaluation to help guide their decisions. With mental health funding consistently failing to meet demand,^30^ it is prudent to quantify whether interventions like CBTp offer good value for money. Cost-effectiveness analysis (CEA) provides a framework for determining whether interventions offer value for money by comparing costs and consequences of alternative treatments. In EIP, this involves examining whether additional CBTp costs are justified by improved outcomes such as reduced relapses, swifter recovery, and long-term savings.

The primary objective of this study was to evaluate the incremental cost-effectiveness of CBTp added to pharmacotherapy compared with pharmacotherapy alone in UK EIP services. Secondary objectives were (1) to identify key drivers of uncertainty in the cost-effectiveness estimate using deterministic sensitivity analysis; (2) to quantify the probability of CBTp being cost-effective across a range of willingness-to-pay thresholds using probabilistic sensitivity analysis; and (3) to characterize the direct and indirect cost components contributing to the societal costs under each treatment strategy. The guiding research question was: In adults with FEP under UK EIP services, is the addition of CBTp to pharmacotherapy cost-effective compared with pharmacotherapy alone, from a societal perspective over a 3-year time horizon, as measured by the incremental cost per QALY gained? Thus, this study provides a novel decision-analytic model informing decisions about EIP resource allocation at a time of sustained funding pressures.

## METHODS

### Methods Overview

A model-based economic evaluation was conducted to compare CBTp +TAU with TAU (pharmacotherapy alone) in EIP. Using a CEA approach, interventions were assessed in terms of their monetary costs and QALY gains. The conceptual model was iteratively simplified to match epidemiological evidence while maintaining face validity. Epidemiological inputs were identified through a targeted literature review. TreeAge Pro Healthcare 2025 version software (TreeAge Software, LLC) was used for model construction and analysis. This study is reported in accordance with Consolidated Health Economic Evaluation Reporting Standards (CHEERS) guidelines.^31^ Additional methodological details have been published previously^32^ and are shown in the **Supplementary Material**.

### Literature Review

A targeted literature review identified inputs for the model. The search strategy aimed to capture effectiveness and economic evidence of CBTp and pharmacotherapy. PubMed, PsycINFO, and the Cochrane Library were searched from inception to June 2025 using controlled vocabulary and free-text terms for early psychosis, CBTp, antipsychotic pharmacotherapy, health-related quality of life and cost-effectiveness. Grey literature, including health technology assessment reports, clinical guidelines, government publications, and reference lists of relevant systematic reviews were also screened. Evidence selection prioritized meta-analyses and systematic reviews, followed by primary UK studies, then international literature. Detailed search and selection criteria are reported in the **Supplementary Material.**

### Model Design, Structure and Mathematical Formulation

Markov models reflect complex disease courses as a series of transitions between mutually exclusive health states over a series of cycles. Costs and effects are incorporated as a mean value per state per cycle. Then, expected values are calculated by adding the costs and outcomes across states and weighted according to the time patients spend in each state.^33^

**Figure 1.**
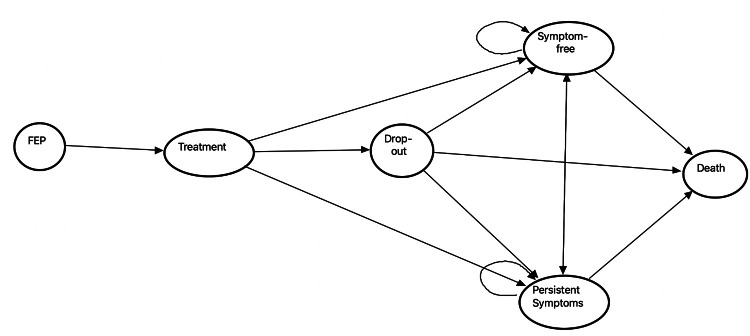
Markov Model State Transition Diagram

A Markov model was developed to compare CBTp  +TAU with TAU alone in a hypothetical FEP cohort. The model simulated disease course through Markov health states, following initial FEP presentation: (1) remission/symptom free; (2) disease/persistent symptoms; (3) death. Recurrent and chronic psychosis states were collapsed into the “persistent symptoms” state because robust evidence to parameterize additional transitions was lacking and the evaluation focused on the early intervention period. From FEP, patients could move to remission, persistent symptoms, or death. Thereafter, they could remain in their current state, relapse from remission to persistent symptoms, or die (where death was modeled as an irreversible absorbing state). A schematic is shown in **Figure 1**.

The model schematic includes 6 states: first episode psychosis (FEP), treatment, dropout, symptom-free, persistent symptoms, and death. In the model patients start in the FEP state and enter treatment. From treatment, patients can either drop out, become symptom-free, develop persistent symptoms, or die. The model allows for transitions between symptom-free and persistent symptom states, with both states having possible transitions to death. Transition probabilities are derived from clinical trial data and published literature. The absorbing death state represents mortality from all causes.

Psychiatrist input ensured the comparators reasonably reflected the NHS model of EIP. Dropouts occurred due to side effects or patient preference at literature-derived probabilities. CBTp was only delivered in conjunction with TAU. In the United Kingdom, prescribing of SGAs predominates,^34^ and evidence suggests that while these drugs differ in their costs, they have similar effectiveness.^35^ Treatment was provided to patients at first presentation and following any relapses. CBTp took the format of 16 one-hour sessions, delivered in-person weekly by a clinical psychologist. No patients in the model were treated with clozapine. Clozapine was excluded as it is reserved for treatment-resistant cases outside the scope of EIP.^36^

### Study Population

The modeled cohort is representative of adults presenting to NHS EIP services with FEP, being majority male with a mean age of presentation in the mid-20s. Patients with psychosis secondary to medical conditions, substance intoxication, or intellectual disability were excluded. The model did not stratify by demographic or clinical subgroups (such as sex, age, ethnicity, socioeconomic deprivation, or comorbidity) owing to insufficient subgroup-level data to parametrize separate transition probabilities. All patients within a given health state were therefore assumed to be homogeneous with respect to costs and outcomes. This is acknowledged as a limitation of the analysis and is discussed below.

### Perspective and Time Horizon

This CEA adopted the societal perspective to capture the comprehensive economic burden of FEP, reflecting how the impacts of psychosis extend beyond direct costs to the healthcare system. The indirect cost components captured under the societal perspective in the model included: lost employment productivity; informal caregiver productivity losses; healthcare system overheads, including indirect work and capital overheads; and patient travel costs to appointments. Stigma-related economic impacts and criminal justice or social service costs were not captured due to the absence of reliable FEP-specific estimates, and their exclusion likely results in a conservative estimate of the true societal burden. Full derivation and assumptions for the indirect cost components are reported in **Supplementary Section S6** and **Supplementary Table S4**.

Due to the often relapsing-remitting nature of psychotic disorders, a multiyear time horizon was necessary to capture downstream costs. Clinical evidence on the incidence and prevalence of disease stages is furthermore often reported in time intervals of 1 year. However, interventions in EIP usually last no longer than 3 months, and patients usually only remain under the care of EIP for a maximum of 3 years. The time horizon of 3 years, divided into 12 three-month cycles, was deemed long enough to capture the costs and effects of the interventions targeting FEP while remaining within the remit of early intervention. Costs and effects were discounted at an annual rate of 3.5%, and half-cycle correction was applied.

### Clinical Outcome Parameters

Transition probabilities were primarily derived from Mayer et al,^13^ a meta-analysis of 28 studies including 2407 FEP participants, as this was the most recent and comprehensive study available. Overall, Mayer et al^13^ concluded that FEP patients may benefit from the addition of CBTp; however, there was a low certainty of the evidence, and no statistically significant difference was found between CBTp added to TAU vs controls for several outcomes including relapses and dropouts. Key transitions included FEP to symptom-free (CBTp, 0.615; TAU, 0.417), FEP to persistent symptoms (CBTp, 0.174; TAU, 0.336), and dropout rates (CBTp, 0.198; TAU, 0.237). Additional parameters were obtained from Wijnen et al^37^ and Robinson et al.^38^ Annual probabilities were converted to 3-month cycle probabilities using standard methods.

To account for uncertainty, the beta distribution with a standard deviation of 30% of the mean was applied to each base case parameter. Key parameter values for each treatment pathway in the model are presented in **Table 1**. Full derivation of all the model parameters is reported in the **Supplementary Material.**

**Table 1.** Key Model Input Parameters

**Parameter**	**CBTp + TAU**	**TAU Alone**	**SD^a^**	**Distribution**	**Source**
Transition probabilities: FEP to next state					
Symptom-free	0.615	0.417	0.185 / 0.125	Beta	^13^
Disease	0.174	0.336	0.052 / 0.101	Beta	^13^
Drop-out	0.198	0.237	0.059 / 0.071	Beta	^13^
Death	0.013	0.010	0.004 / 0.003	Beta	^13^
Transition probabilities: Disease state (both arms)^b^					
Remission	0.350		0.105	Beta	^37^
Persistent symptoms	0.636		0.191	Beta	^37^
Death	0.014		0.004	Beta	^37^
Transition probabilities: Dropout state					
Symptom-free	0.506	0.110	0.152 / 0.033	Beta	^38^
Persistent symptoms	0.438	0.834	0.131 / 0.250	Beta	^38^
Death	0.056		0.017	Beta	^38^
Utility values (both arms)					
Remission/symptom-free	0.756		0.227	Beta	^37^
Disease/persistent symptoms	0.362		0.109	Beta	^37^
Death	0		—	—	^—^
Direct costs, annual (£)					
Olanzapine 20 mg/day	334.60		100.38	Gamma	^39^
Aripiprazole 10 mg/day	231.00		69.30	Gamma	^39^
Risperidone 4 mg/day	147.00		44.10	Gamma	^39^
CBTp (16 sessions)	591.68		117.50	Gamma	^40^
Healthcare monitoring	562.26		168.68	Gamma	^41^
Indirect costs, annual (£)					
Indirect work — psychologist	394.45		118.34	Gamma	^40^
Indirect work — psychiatrist	738.02		221.41	Gamma	^40^
Overheads — psychologist	266.26		79.88	Gamma	^40^
Overheads — psychiatrist	207.57		62.27	Gamma	^40^
Capital overheads	3191.00		957.30	Gamma	^40^
Non-staff costs (informal care)	17 520.00		5256.00	Gamma	^40^
Travel to appointment	110.00		33.00	Gamma	^42^
Lost employment	28 918.00		8675.40	Gamma	^43^

Abbreviations: CBTp, cognitive behavioral therapy for psychosis; SD, standard deviation; TAU, treatment as usual.

^a^Where 2 SD values are shown (x/y), x corresponds to CBTp + TAU and y to TAU alone.

^b^Disease-state transitions are assumed equal across both arms, reflecting the hypothesis that progression once established is independent of initial treatment allocation.

### Utility Parameters

Health-state utilities were used to calculate QALYs by multiplying the time spent in each state by its associated utility weight. The values were obtained from Wijnen et al^37^: remission/symptom-free (0.756) and disease/persistent symptoms (0.362). Death was assigned a utility of zero. Beta distributions with a standard deviation of 30% of the mean were applied to capture uncertainty and patient-level variation.

### Cost Parameters

Cost parameters were primarily obtained from the Personal Social Services Research Unit (PSSRU),^40^ NHS Agenda for Change pay bands,^44^ and NICE guidance.^24^ All direct costs were reported in 2025 British pounds, with inflation adjustment using the NHS cost uplift factor.^45^

The direct costing framework followed a microcosting approach supplemented by gross costing using national references where detailed resource use data were unavailable. The direct costs incorporated in this model included costs of consultant psychiatrist reviews and regular blood tests and monitoring, in addition to drug costs and the cost of CBTp administration. Consultant psychiatrist reviews were included as these represent the primary medical oversight cost within EIP.

Labor costs were calculated using estimated salary ranges for publicly funded clinicians, based on published data, job postings, and clinician consultation. The salary for a clinical psychologist in England is approximately £46 148,^46^ and the salary for a consultant general psychiatrist approximately £123 000.^47^ To obtain labor costs directly associated with clinical activities, an applied hourly rate was calculated based on these salary estimates. Applied hourly rates represented the time clinicians spend on nonpatient activities,^48^ assuming a 60% direct work for psychologists and 1:1.6 direct-to-indirect ratio for psychiatrists.^40^

Drug costs were obtained from the British National Formulary^39^ and June 2025 edition of the NHS Business Services Authority data on generic prescriptions.^49^ Three SGAs were modeled: olanzapine, aripiprazole, and risperidone, resulting in annual per-patient drug costs ranging from £147.00 to £334.60, which were averaged in the model. These 3 agents were selected as they represent the most commonly prescribed SGAs in UK EIP settings, ensuring the modeled drug costs reflect routine clinical practice. The costs for blood tests and pathology assumed the patient would need a twice-yearly comprehensive metabolic panel. This is consistent with NICE guidance on metabolic monitoring for patients initiated on SGAs, reflecting the known cardiometabolic risks associated with these drugs.^24,39^

Costs not modeled included GP assessment, hospitalization, and social service provision, as these are not systematically incurred within standard EIP pathways or lacked robust FEP-specific estimates.

Indirect costs represent the total economic burden of a disease to society. Each indirect cost component in this model was included based on empirical evidence that FEP disproportionately affects young adults during early employment years, generates substantial informal care demands, and imposes overhead costs on healthcare providers delivering mental health services.^50^ Indirect costs were calculated using a simplified version of the human capital framework outlined by Bonin,^51^ prioritizing components with the strongest empirical foundations given the dearth of literature on presenteeism, educational attainment, and long-term welfare dependency in FEP. These simplifications were necessary to maintain analytical tractability while preserving the key drivers of any cost-effectiveness conclusions.

For the healthcare system, the costs of overheads and indirect work were considered, referring to activities that support patient care but do not involve physical interaction with the patient. Indirect societal costs included the costs of travel to appointments, the costs of informal care, and lost employment productivity. Quantifying the indirect costs of absenteeism was based on the median salary of 22- to 29-year-olds in the UK.^43^ Informal caregiver productivity losses were calculated by assigning a monetary value to unpaid care based on average hours of support for FEP patients and national wage estimates.

### Model Validation

The model was validated through clinician review for face and operational validity, ensuring comparators reflected NHS EIP practice and outcomes fell within plausible ranges. Cross-validation confirmed consistency with prior research. Model fit testing was not applicable because none of the parameters were regression-derived.

### Economic Evaluation

Cost-effectiveness was determined using the incremental cost-effectiveness ratio (ICER), calculated as the additional cost per additional QALY gained when adding CBTp to TAU. The analysis considered NICE’s willingness-to-pay (WTP) range of £20 000 to £30 000 per QALY gained.^52^ NICE does not stipulate a maximum WTP threshold or ICER but generally considers an ICER of less than £20 000 per QALY gained to be cost-effective.^52^ Between £20 000 and £30000, NICE recommends considering additional factors that may mean the ICER underestimates cost-effectiveness.^53^

### Sensitivity Analysis

Deterministic sensitivity analysis was performed to identify the model parameters with the greatest influence on the ICER. Results were presented as a tornado diagram.

Probabilistic sensitivity analysis was performed to generate the most accurate estimate of the ICER and account for parameter uncertainty. Monte Carlo simulation with 100 000 iterations propagated parameter uncertainty using appropriate distributions: beta distribution for utilities and transition probabilities, and gamma distribution for costs. Results were presented with the cost-effectiveness acceptability curves and incremental cost-effectiveness scatterplot.

## RESULTS

### Base Case Cost-Effectiveness Analysis

Base case analysis demonstrated that adding CBTp to standard pharmacotherapy (TAU) in EIP generated modest health benefits at incremental cost near conventional cost-effectiveness thresholds. Total costs per patient were £52 736.77 for CBTp +TAU and £51 484.38 for TAU alone, yielding an incremental cost of £1252.39.

**Figure 2.**
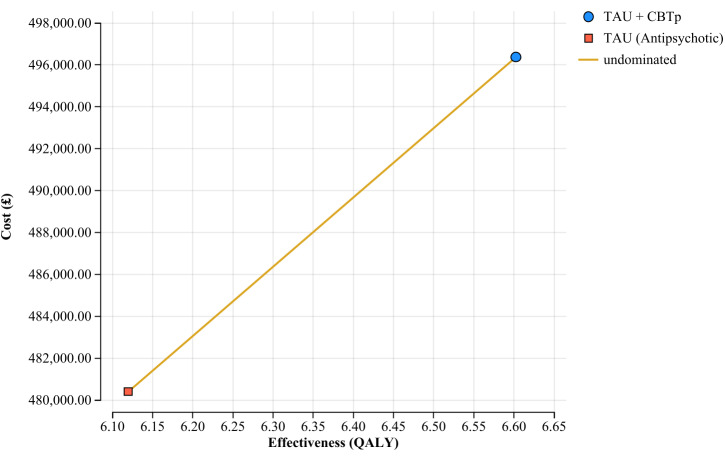
Cost-Effectiveness Frontier of TAU + CBTp vs TAU Abbreviations: CBTp, cognitive behavioral therapy for psychosis; QALY, quality-adjusted life-year; TAU, treatment as usual.

CBTp +TAU generated 6.60 QALYs compared with 6.12 QALYs for TAU alone (incremental QALYs, 0.48), resulting in an ICER of £32 979 per QALY gained. This exceeds NICE’s upper WTP threshold of £30 000 per QALY. The cost-effectiveness frontier (**Figure 2**) showed both treatments were undominated. At WTP thresholds below £32 979 per QALY, TAU would be preferred; above this, CBTp +TAU was preferred.

The gold line is the cost-effectiveness frontier which connects the undominated treatment options. CBTp+TAU is positioned to the upper-right of TAU, indicating it provides greater effectiveness at higher cost. The gradient represents the incremental cost-effectiveness ratio between the 2 interventions, showing the cost per QALY gained when moving from TAU to TAU + CBTp.

### Deterministic Sensitivity Analysis

**Figure 3.**
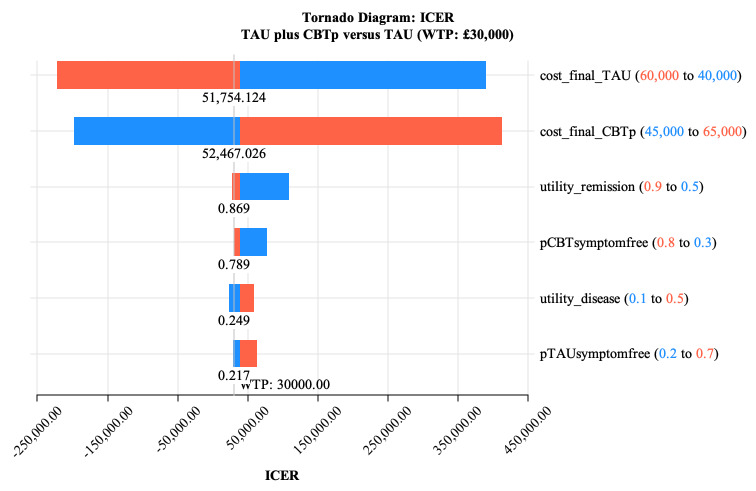
Tornado Diagram Abbreviations: CBTp, cognitive behavioral therapy for psychosis; cost_final_TAU, total cost of TAU; cost_final_CBTp, total cost of CBTp + TAU; ICER, incremental cost-effectiveness ratio; utility_remission, utility value for remission state; utility_disease: utility value for disease state; pTAUsymptomfree: probability of achieving symptom-free status following TAU; pCBTsymptomfree: probability of achieving symptom-free status following CBTp with TAU; TAU, treatment as usual.

Deterministic sensitivity analysis revealed cost parameters as the dominant sources of uncertainty (**Figure 3**). TAU costs showed the largest influence, with ICERs spanning from approximately –100 000 (cost-saving) to +£200 000 per QALY. Higher TAU costs generated strongly negative ICERs, while lower costs pushed ICERs far above the £30 000 threshold. CBTp costs demonstrated inverse influence, with higher costs increasing the ICER.

Each horizontal bar represents the range of ICER values at a willingness-to-pay threshold of £30 000 per QALY when a parameter is varied between its lower and upper bounds while holding all other parameters at their base case values. The blue portion of the bar positioned left of the red portion indicates the ICER increased as the parameter value increased. The vertical line at £30 000 represents the willingness-to-pay threshold. Parameters are ranked by their impact on the ICER, with final costs for both TAU (varied from £40 000 to £60 000) and CBTp (varied from £45 000 to £65 000) having the greatest influence on cost-effectiveness. The base case model inputs are shown in the center. The diagram demonstrates that cost parameters have substantially more impact on the ICER than effectiveness parameters.

The utility value for the remission/symptom-free state was the next most influential parameter, with ICERs ranging from £25 000 to £110 000 per QALY. Higher utility values generated more favorable ICERs, with values above 0.869 positioning CBTp below the £30 000 threshold. The probability of achieving symptom-free status after CBTp showed moderate influence, while disease state utility and TAU symptom-free probability demonstrated minor effects.

### Probabilistic Sensitivity Analysis

**Figure 4.**
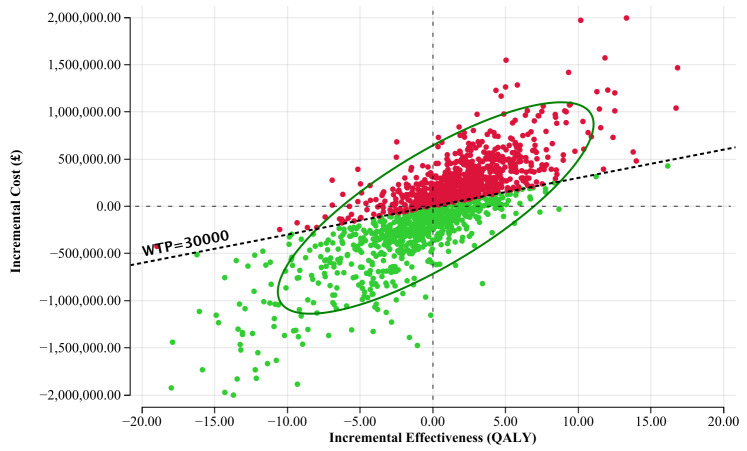
Incremental Cost-Effectiveness Scatterplot of TAU + CBPp vs TAU (Antipsychotic) Abbreviations: CBTp, cognitive behavioral therapy for psychosis; QALY, quality-adjusted life-year; TAU, treatment as usual; WTP, willingness to pay.

A Monte Carlo simulation with 100 000 iterations demonstrated uncertainty around base case estimates. The incremental cost-effectiveness scatterplot (**Figure 4**) showed wide dispersion across quadrants, with substantial proportions in both top-right (more effective, more costly) and bottom-left (less effective, less costly) quadrants. Few fell in the bottom-right quadrant (more effective, less costly). The 95% confidence ellipse spanning both sides of the WTP line illustrated the marginal cost-effectiveness case. Neither intervention dominated, as neither consistently delivered more QALYs at lower cost.

Each point represents one of the first 1500 Monte Carlo iterations. The diagonal dashed line represents the WTP threshold of £30 000 per QALY. Green points below this line indicate cost-effective scenarios at the WTP threshold. The 95% confidence ellipse encompasses the central distribution of the simulation results, illustrating the uncertainty around the incremental cost-effectiveness ratio.

**Figure 5.**
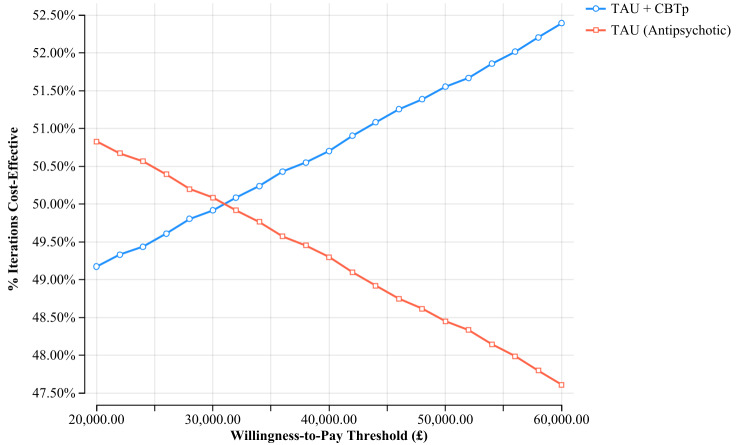
Cost-Effectiveness Acceptability Curve Abbreviations: CBTp, cognitive behavioral therapy for psychosis; TAU, treatment as usual.

The cost-effectiveness acceptability curves (**Figure 5**) demonstrated how the probability of cost-effectiveness in the CBTp group varied with WTP threshold: 49.2% at £20 000 per QALY, 49.9% at £30 000 per QALY, and 50.7% at £40 000 per QALY, placing the intervention near the boundary of cost-effectiveness acceptability.

The cost-effectiveness acceptability curve (CEAC) shows the probability that each intervention is cost-effective across different willingness-to-pay thresholds. The blue line represents TAU + CBTp and the red line represents TAU alone. The curves intersect at approximately £32 000 per QALY, indicating that CBTp + TAU becomes the more cost-effective option above this threshold. At the commonly used UK threshold of £30 000 per QALY, CBTp + TAU has a 49.9% probability of being cost-effective.

## DISCUSSION

### Principal Findings

To the authors’ knowledge, this is the first decision-analytic model evaluating the cost-effectiveness of CBTp within UK EIP services. The base case analysis yielded an ICER of £32 979 per QALY, exceeding NICE’s conventional £30 000 threshold. However, probability sensitivity analysis revealed substantial uncertainty, with CBTp demonstrating cost-effectiveness in approximately 50% of simulated scenarios at the £30 000 WTP threshold. These findings suggest that adding CBTp to standard pharmacotherapy in EIP provides borderline cost-effectiveness with considerable uncertainty over a 3-year horizon.

### Comparison to Existing Literature

The findings largely align with broader literature on CBTp and EIP; however, direct comparisons are complicated by methodological heterogeneity across studies and differences in population, perspective, and time horizon. Previous evaluations found comprehensive EIP likely cost-effective. The systematic review by Aceituno et al^20^ concluded that there is consistent evidence that the implementation of EIP might be cost-effective. Similarly, Rosenheck et al^54^ found the RAISE comprehensive FEP intervention program is likely cost-effective (94% probability), again reporting a bundled rather than component-level analysis. This is substantially higher than the approximately 50% probability of cost-effectiveness found here. The disparity is consistent with the hypothesis that the cost-effectiveness case is stronger for an integrated service package than for any single component added to already-effective pharmacotherapy. The contribution of the present study is therefore to disaggregate the CBTp component specifically, revealing that its incremental value, while plausible, is more uncertain than that of the broader EIP model.

The heterogeneity among studies,^55^ reflected in analysis by Mayer et al,^13^ stems from variations in patient characteristics, therapist competence, service integration, and outcome measures. This study’s societal perspective adds new evidence on medium-term outcomes. The most directly comparable study is the EPiSODe model,^56^ which evaluated CBTp added to TAU in a real-world schizophrenia population using a discrete event simulation. That study found CBTp cost-effective in 61.2% of simulations with an ICER of €12 947 per QALY. This was a more favorable result than the £32 979 per QALY found here, and several factors likely explain this difference. The EPiSODe model used a real Dutch dataset rather than a hypothetical cohort, used a longer simulation horizon, adopted only a healthcare perspective, and excluded indirect costs. Additionally, the population encompassed established schizophrenia spectrum disorders rather than FEP, meaning patients may have had greater baseline service utilization against which CBTp’s cost-offsetting effects were more pronounced. These differences highlight that the cost-effectiveness of CBTp is likely context-dependent, varying with population, service configuration, and analytical perspective. Ising et al,^57^ evaluating the EDIE-NL trial, found that preventive CBT for individuals at ultra-high risk of psychosis had an 83% probability of being more effective and less costly than routine care, with a 92% probability of cost-effectiveness at the Dutch threshold over 4 years. However, this study evaluated CBT for the prevention of FEP rather than its treatment, representing a meaningfully different clinical context. The case for cost-effectiveness in prevention is strengthened by the potential to entirely avert the downstream costs of a first psychotic episode. This cost-offsetting mechanism does not apply after FEP has occurred.

Altogether, the broader literature suggests that CBTp is likely to be cost-effective across a range of psychosis-related contexts, though the magnitude and certainty of this conclusion varies. The present study’s near-50% probability of cost-effectiveness should not be interpreted as evidence against CBTp in EIP. Rather, it reflects inherent limitations in health technology assessment for psychological interventions, including variability in clinical effectiveness, heterogeneous implementation costs, and limited long-term follow-up data.

### Strengths and Limitations

This study had several methodological strengths. The Markov model appropriately simulated the episodic nature of early psychosis through discrete health states reflecting clinical trajectories observed in FEP populations. Because the model tracked patients through 12 cycles, it captured both recurrent psychotic episodes and the potential for sustained recovery. This multi-year perspective is appropriate because immediate benefits may be modest while cumulative effects are substantial. Clinician input was integrated with assumptions from an early stage, increasing the model’s clinical relevance, while comprehensive sensitivity analyses rigorously quantified uncertainty. Disaggregating direct and indirect costs into their specific components improved transparency and precision. The societal perspective captured broader economic impacts beyond healthcare costs, essential given the early onset and substantial indirect costs of FEP.

Evidence limitations constrained the model, with transition probabilities primarily derived from a single meta-analysis^13^ incorporating UK, European, and North American studies. This geographic heterogeneity may not fully represent NHS outcomes. Small sample sizes and incomplete disease stage coverage in available studies introduced uncertainty. The operator-dependent nature of CBT^58^ and the efficacy-effectiveness gap further affect generalizability. The 30% standard deviation applied to probability distributions, while conventional,^33^ was arbitrary. Equivalent CBTp efficacy for FEP, relapses, and persistent symptoms was assumed.

Combining outcomes into single states (relapses/treatment failure into “disease”; all mortality into “death”) enabled modeling despite evidence gaps but sacrificed granularity. Suicide prevention was only captured through general mortality due to insufficient CBTp-specific data. The memoryless Markov assumption, while enabling computational feasibility, overlooks how the duration of untreated psychosis and patient history influence outcomes. Similarly, time in state is not accounted for, and under this assumption those who have had longer periods of remission are not more likely to remain in remission than those returning to remission from relapse.

Cohort-based rather than individual patient microsimulation was necessitated by limited individual-level data but assumed within-state homogeneity. The model did not stratify by demographic or clinical characteristics known to influence FEP outcomes, including sex, age of onset, ethnicity, socioeconomic status, DUP, and baseline symptom severity. While published FEP epidemiology provides descriptive characterization of the study population, insufficient subgroup-level data were available to parameterize demographically stratified transition probabilities. Future modeling incorporating individual patient data or subgrouplevel meta-analytic estimates would allow examination of whether CBTp cost-effectiveness differs across clinically meaningful patient subgroups.

The 3-year horizon, while aligning with typical EIP duration, may conservatively estimate the long-term value of CBTp by not capturing lifetime benefits. Extrapolating beyond this time frame would introduce more uncertainty given sparse empirical long-term data. Analytical robustness over speculative projections was prioritized.

National tariffs and PSSRU unit costs,^40^ while standardized, may not reflect true economic costs or capture variation across services, locations, and patient complexity. Estimating CBTp delivery costs required assumptions about session frequency and staffing that may not reflect real-world implementation and excluded one-off training costs. Indirect cost valuation, particularly productivity losses using the human capital approach, may overestimate impacts for young patients with limited employment history.^51^ Furthermore, the societal perspective used in this model, while comprehensive relative to healthcare-only analyses, did not capture stigma-related economic impacts, criminal justice costs, or social service provision, meaning the true indirect burden of FEP is likely underestimated. These limitations in costing approach mean financial estimates should be interpreted as approximations, with the true cost-effectiveness of CBTp potentially varying significantly across different EIP contexts.

Focusing exclusively on CBTp plus pharmacotherapy while excluding family interventions, vocational rehabilitation, and social programs was necessary for analytical clarity but may underestimate the value of comprehensive EIP and overestimate CBTp’s incremental benefit by not accounting for diminishing returns within broader service packages. Using QALYs as the outcome measure, while enabling standardized comparison, may inadequately capture social functioning, educational attainment, and employment improvements central to recovery-oriented care,^59^ and poorly reflect caregiver burden.^60^ Nevertheless, QALYs remain justified as the standard metric for health technology assessment, ensuring policy relevance and cross-intervention comparability.

### Clinical and Policy Implications

The uncertainty presents differential implications across stakeholders. Healthcare commissioners and policymakers face lower risk tolerance given resource constraints and the opportunity costs of funding interventions with uncertain cost-effectiveness. Conversely, clinicians and patients may accept higher risk given limited treatment alternatives and the potentially catastrophic consequences of inadequate intervention. Implementation reversibility varies. At the service level, establishing CBTp programs requires substantial upfront investment in staff and service configuration that cannot be easily reversed quickly, creating path dependency. Individual treatment decisions retain flexibility for discontinuation if benefits are not seen.

Overall, the findings provide cautious support for CBTp implementation in EIP. First, the base case demonstrated health benefits through QALY gains when CBTp is effective. Second, the societal perspective captured broader economic benefits representing genuine societal value beyond direct costs. Third, this analysis suggests NICE’s existing CBTp recommendation for FEP^24^ is economically justified, albeit with significant uncertainty. Sensitivity analyses revealed the model was most responsive to cost and remission-state utility parameters, suggesting that targeted improvements to make CBTp delivery more efficient could tip the balance toward consistent cost-effectiveness. Incremental implementation strategies—pilot programs or phased rollouts targeting patients most likely to benefit—may be most appropriate.

These findings primarily apply to high-income settings. Data derived from European and North American studies limit applicability to low- and middle-income countries, where implementation discussions must balance CBT’s clinical effectiveness against more basic healthcare service gaps. This is fundamentally a policy question requiring robust cost-effectiveness evidence aligned with specific population needs.

### Future Research Implications

These results highlight several research priorities. Large-scale, long-duration RCTs on CBTp with embedded economic evaluations would reduce uncertainty in clinical and cost-effectiveness estimates. Research identifying patient characteristics and service factors associated with optimal CBTp outcomes would enable stratified implementation and improve real-world cost-effectiveness. Such policies would need to be evaluated in their own right; it is possible that costs in the subgroups most likely to benefit are also higher, thus it is not necessarily predictable how these changes would impact cost-effectiveness. Implementation research comparing delivery models (eg, individual vs group therapy, face-to-face vs digital delivery) could identify optimal approaches. Future evaluations should also examine other EIP components including family interventions and social programs to inform comprehensive service configuration decisions.

The results of this study are significant given budget restraints within the NHS^61^ and the criticism EIP has received since its inception.^62^ Further research with longer follow-up and diverse populations would reduce uncertainty and optimize EIP service configuration during the critical early intervention period.

## CONCLUSIONS

This cost-effectiveness analysis found that CBTp as an adjunct to pharmacotherapy in UK EIP services achieves borderline cost-effectiveness, being cost-effective in approximately 50% of simulations at the £30,000 WTP threshold (base case ICER, £32,979 per QALY). The uncertainty reflects heterogeneity in treatment response, varying illness severity across studies, and challenges capturing psychological intervention effects within conventional economic frameworks, rather than evidence against CBTp cost-effectiveness.

These findings support cautious CBTp implementation in EIP. Targeted approaches considering patient characteristics, delivery models, and local contexts may be more appropriate than blanket policies. The near-threshold positioning indicates that under certain conditions CBTp may represent good value for money. This study thus provides valuable economic evidence supporting NICE’s current recommendation while highlighting priorities for ongoing evaluation as EIP services evolve.

### Disclosures

The authors report no conflicts of interest.

## Supplementary Material

Online Supplementary Material

## References

[1] American Psychiatric Association (2013). Diagnostic and Statistical Manual of Mental Disorders.

[2] Breitborde N. J., Srihari V. H., Woods S. W. (2009). Review of the operational definition for first-episode psychosis. Early Interv Psychiatry.

[3] Hodgekins J., Birchwood M., Christopher R.. (2015). Investigating trajectories of social recovery in individuals with first-episode psychosis: a latent class growth analysis. Br J Psychiatry.

[4] Simon G. E., Coleman K. J., Yarborough B. J. H.. (2017). First presentation with psychotic symptoms in a population-based sample. Psychiatr Serv.

[5] Morgan C., Dazzan P., Lappin J.. (2021). Rethinking the course of psychotic disorders: modelling long-term symptom trajectories. Psychol Med.

[6] Serra-Arumí C., Golay P., Bonnarel V.. (2023). Risk and protective factors for recovery at 3-year follow-up after first-episode psychosis onset: a multivariate outcome approach. Soc Psychiatry Psychiatr Epidemiol.

[7] Jardri R. (2013). Brain imaging of first-episode psychosis [article in French]. Encephale.

[8] Inchausti L., Gorostiza I., Gonzalez Torres M. A., Oraa R. (2023). The transition to schizophrenia spectrum disorder from a first psychotic episode that did or did not appear to be induced by substance use. Psychiatry Res.

[9] Lin D., Kim H., Wada K.. (2022). Unemployment, homelessness, and other societal outcomes in patients with schizophrenia: a real-world retrospective cohort study of the United States Veterans Health Administration database. BMC Psychiatry.

[10] NHS Transformation Directorate Early intervention in psychosis.

[11] Jin H., Mosweu I. (2017). The societal cost of schizophrenia: a systematic review. Pharmacoeconomics.

[12] Correll C., Solmi M., Croatto G.. (2022). Mortality in people with schizophrenia: a systematic review and meta-analysis of relative risk and aggravating or attenuating factors. World Psychiatry.

[13] Mayer S. F., Corcoran C., Kennedy L., Leucht S., Bighelli I. Cognitive behavioural therapy added to standard care for first-episode and recent-onset psychosis. Cochrane Database Syst Rev.

[14] Correll C. U., Galling B., Pawar A.. (2018). Comparison of early intervention services vs treatment as usual for early-phase psychosis: a systematic review, meta-analysis, and meta-regression. JAMA Psychiatry.

[15] Lambert M., Schöttle D., Sengutta M.. (2015). Early detection and integrated care in adolescents and young adults with severe psychotic illnesses. Psychiatr Prax.

[16] Williams R., Ostinelli E. G., Agorinya J.. (2024). Comparing interventions for early psychosis: a systematic review and component network meta-analysis. EClinicalMedicine.

[17] Marshall M., Rathbone J. (2011). Early intervention for psychosis. Cochrane Database Syst Rev.

[18] Goldner-Vukov M., Cupina D. D., Moore L. J., Baba-Milkic N., Milovanovic S. (2007). Early intervention in first episode psychosis: hope for a better future. Srp Arh Celok Lek.

[19] Tsiachristas A., Thomas T., Leal J., Lennox B. R. (2016). Economic impact of early intervention in psychosis services: results from a longitudinal retrospective controlled study in England. BMJ Open.

[20] Aceituno D., Vera N., Prina A. M., McCrone P. (2019). Cost-effectiveness of early intervention in psychosis: systematic review. Br J Psychiatry.

[21] Ceraso A., Lin J. J., Schneider-Thoma J.. (2020). Maintenance treatment with antipsychotic drugs for schizophrenia. Cochrane Database Syst Rev.

[22] Woodall A. (2021). Managing patients on atypical antipsychotics in primary care. Prescriber.

[23] Smith T., Weston C., Lieberman J. (2010). Schizophrenia (maintenance treatment). Am Fam Physician.

[24] National Institute for Health and Care Excellence (2014). Psychosis and Schizophrenia in Adults: Prevention and Management.

[25] Chand S.P., Kuckel D.P., Huecker M.R. (2023). StatPearls.

[26] Hagen R. (2013). CBT for Psychosis: A Symptom-Based Approach.

[27] Health Quality Ontario (2018). Cognitive behavioural therapy for psychosis: a health technology assessment. Ont Health Technol Assess Ser.

[28] Agbor C., Kaur G., Soomro F. M.. (2022). The role of cognitive behavioral therapy in the management of psychosis. Cureus.

[29] Sheaves B., Peters E., Stahl D., Johns L. (2020). Changes in care costs associated with cognitive behavioural therapy for psychosis delivered in routine clinical practice. J Ment Health.

[30] Gilburt H., Mallorie S. (2024). Mental health 360: funding and costs.

[31] Husereau D., Drummond M., Augustovski F.. (2022). Consolidated Health Economic Evaluation Reporting Standards 2022 (CHEERS 2022): explanation and elaboration: a report of the ISPOR CHEERS II Good Practices Task Force. Value Health.

[32] Duckworth M. (2025). Cognitive Behavioural Therapy in Early Intervention for Psychosis: A Cost-Effectiveness Analysis.

[33] Briggs A., Sculpher M., Claxton K. (2006). Decision Modelling for Health Economic Evaluation.

[34] Marston L., Nazareth I., Petersen I., Walters K., Osborn D.P. (2014). Prescribing of antipsychotics in UK primary care: a cohort study. BMJ Open.

[35] Hamina A., Taipale H., Lieslehto J.. (2024). Comparative effectiveness of antipsychotics in patients with schizophrenia spectrum disorder. JAMA Netw Open.

[36] Nucifora F. C., Jr., Mihaljevic M., Lee B. J., Sawa A. (2017). Clozapine as a model for antipsychotic development. Neurotherapeutics.

[37] Wijnen B. F. M., Thielen F. W., Konings S.. (2020). Designing and testing of a health-economic Markov model for prevention and treatment of early psychosis. Exp Rev Pharmacoecon Outcomes Res.

[38] Robinson D., Woerner M. G., Alvir J. M.. (1999). Predictors of relapse following response from a first episode of schizophrenia or schizoaffective disorder. Arch Gen Psychiatry.

[39] Joint Formulary Committee (2025). British National Formulary.

[40] Dargan A. (2024). Unit Costs of Health and Social Care 2023 Manual.

[41] National Institute for Health and Care Excellence (2016). NG45: Routine Preoperative Tests for Elective Surgery – Guideline Appendices 1–3.

[42] Stagecoach East (2025). Never pay more than £3 for your single journey.

[43] Office for National Statistics (2024). Earnings and hours worked, age group: ASHE Table 6.

[44] NHS Health Careers (2025). Agenda for Change – pay rates.

[45] NHS England (2025). Revenue finance and contracting guidance for 2025–26.

[46] Prospects (2025). Job profile: clinical psychologist.

[47] NHS Health Careers (2025). Pay for doctors.

[48] Gidwani R. A. (2009). A Budget Impact Analysis of Chronic Disease Screening: The Business Case of Rapid HIV Testing in VA Emergency Departments.

[49] NHS Business Services Authority (2025). NHS electronic drug tariff.

[50] Koopmanschap M. A., Rutten F. F. (1993). Indirect costs in economic studies: confronting the confusion. Pharmacoeconomics.

[51] Bonin E. M. (2017). The Societal Costs of Anorexia Nervosa in England.

[52] National Institute for Health and Care Excellence (2022). NICE Health Technology Evaluations: The Manual.

[53] McCabe C., Claxton K., Culyer A. J. (2008). The NICE cost-effectiveness threshold: what it is and what that means. Pharmacoeconomics.

[54] Rosenheck R., Leslie D., Sint K.. (2016). Cost-effectiveness of comprehensive, integrated care for first episode psychosis in the NIMH RAISE Early Treatment Program. Schizophr Bull.

[55] Tarrier N. (2014). CBT for psychosis: effectiveness, diversity, dissemination, politics, the future and technology. World Psychiatry.

[56] Konings S. R. A., Berkhof M., Veling W.. (2025). Cognitive behavioral therapy for psychosis: a cost-effectiveness study using the EPiSODe model. Eur Psychiatry.

[57] Ising H. K., Lokkerbol J., Rietdijk J.. (2017). Four-year cost-effectiveness of cognitive behavior therapy for preventing first-episode psychosis: the Dutch Early Detection Intervention Evaluation (EDIE-NL) trial. Schizophr Bull.

[58] Ruggiero G. M., Spada M. M., Caselli G., Sassaroli S. (2018). A historical and theoretical review of cognitive behavioral therapies: from structural self-knowledge to functional processes. J Ration Emot Cogn Behav Ther.

[59] Dawoud D., Lamb A., Moore A.. (2022). Capturing what matters: updating NICE methods guidance on measuring and valuing health. Qual Life Res.

[60] NICE Decision Support Unit Carer health related quality of life.

[61] Jefferies D., Wickens C. (2024). Tight budgets and tough choices.

[62] Jewell M., Lord R., Sarfraz M. A. (2023). Model of care in early intervention in psychosis: how do we make the square peg rounder?.

