# Supplementary material for: Cognitive Behavioral Therapy in Early Intervention for Psychosis: A Cost-Effectiveness Analysis
Source: J Health Econ Outcomes Res. 2026 Jun 11;13(1):162047. doi: 10.36469/001c.162047 (PMC13264041; doi:10.36469/001c.162047)
Supplement: Online Supplementary Material [file jheor_2026_13_1_162047_348142.pdf]

Journal of Health Economics and Outcomes Research

## Online Supplementary Material

Cognitive Behavioral Therapy in Early Intervention for Psychosis: A Cost-Effectiveness Analysis. *JHEOR*. 2026;13(1):??-??. doi:10.36469/jheor.2026.162047

**S1: Literature Review and Evidence Selection** ........ 1

**S2: Model Overview** ........ 2

**S3. Clinical Outcome Parameters** ........ 3

**S4: Utility Parameters** ........ 6

**S5: Direct Cost Parameters and Costing Assumptions** ........ 6

**S6: Indirect Cost Parameters and Societal Perspective** ........ 9

**S7: Economic Evaluation** ........ 12

**S8: Deterministic Sensitivity Analysis** ........ 12

**S9: Probabilistic Sensitivity Analysis – Technical Details** ........ 13

**References** ........ 14

This supplementary material has been provided by the authors to give readers additional information about their work.

This is an open-access article distributed under the terms of the Creative Commons Attribution 4.0 International License (CCBY-4.0). View this license's legal deed at http://creativecommons.org/licenses/by/4.0 and legal code at http://creativecommons.org/licenses/by/4.0/legalcode for more information.

## S1. Literature Review and Evidence Selection

A targeted literature review was conducted to identify model inputs. The search strategy was designed to capture both clinical effectiveness and economic evidence relevant to CBT for psychosis (CBTp) and pharmacotherapy.

Searches were run in PubMed, PsycINFO and the Cochrane Library from database inception to June 2025. A combination of controlled vocabulary (e.g., MeSH headings) and free-text terms was used. Search strings included variations of terms such as:

- "first episode psychosis", "FEP", "early psychosis", "early intervention in psychosis", "EIP"
- "cognitive behavioural therapy for psychosis", "CBTp", "cognitive behaviour therapy", "psychological therapy"
- "antipsychotic drug", "second-generation antipsychotic", "SGA", "olanzapine", "aripiprazole", "risperidone"
- "health-related quality of life", "HRQoL", "EQ-5D", "health utility"
- "cost-effectiveness", "economic evaluation", "cost–utility", "transition probability".

Search terms were adapted to each database's indexing system. In addition to database searches, grey literature was screened, including health technology assessment (HTA) reports, clinical guidelines and government publications relevant to psychosis care and EIP models. Reference lists of key systematic reviews and primary studies were manually screened, and forward citation searches were conducted on pivotal CBTp and FEP trials.

Studies with a follow-up duration of at least 12 months were preferred to capture medium-term course. Data extraction focused on key model parameters: relapse rates, recovery probabilities, drop-out rates, mortality, prevalence ratios between health states and health-related quality of life values. Where direct FEP-specific estim1ates were unavailable,

data from broader psychosis populations were used and adjusted. Full search strings and inclusion/exclusion criteria can be provided on request.

## S2. Model Overview

The model compared CBTp plus treatment as usual (CBTp+TAU) with TAU alone in a hypothetical cohort of adults with diagnosed first-episode psychosis (FEP) managed in NHS EIP services. Following initial FEP presentation, individuals could occupy one of three mutually exclusive health states:

1. Remission/symptom-free
2. Disease/persistent symptoms
3. Death

States representing recurrence, relapse, multiple relapses and chronic psychosis were collapsed into the single "persistent symptoms" state. This simplification was adopted because
(a) there was insufficient robust evidence to parametrise transitions between multiple recurrent/chronic states, and (b) the evaluation specifically targeted the early-intervention period rather than the entire long-term psychosis trajectory. Including additional long-term psychosis states would have increased model complexity without meaningfully contributing to the research question.

At model entry, patients were in the FEP state and progressed through one of two treatment arms: CBTp+TAU or TAU (pharmacotherapy alone). From FEP, patients could transition to remission, persistent symptoms or death. From remission, they could remain

in remission, relapse to persistent symptoms or die. From persistent symptoms, they could remain symptomatic, remit or die. Death was modelled as an absorbing state from which no further transitions were possible.

Expert consultation ensured that the model structure and assumptions reflected routine NHS EIP practice for CBTp and pharmacotherapy. TAU pharmacotherapy was simplified, and drop-out was modelled using probabilities derived from the literature, capturing both withdrawal due to severe side-effects and patient preference.

The model followed the Markov property, assuming that transition probabilities depended only on the current health state and not on the prior trajectory. Formally:

$$P(X_{t+1} = j \mid X_t = i, X_{t-1} = i_{t-1}, \ldots, X_0 = i_0) = P(X_{t+1} = j \mid X_t = i)$$

where the probability of moving to the next state $j$ depends only on the current state $i$ at time $t$ and not on any of the previous states. At the end of each cycle, transitions were governed by a transition probability matrix P, where $Pij$ denotes the probability of moving from state i to state j in one cycle. This formulation assumes that all relevant prognostic information is captured within the current health state and that the future trajectory is independent of how patients arrived there. The Markov probability tree illustrating possible transitions over a single cycle is provided in **Figure S1.**

## S3. Clinical Outcome Parameters

Relapse, defined as treatment failure and recurrence of acute psychosis, is common in FEP and chronic psychotic disorders, with up to 80% of FEP patients relapsing within five years[1]. To estimate the incremental cost-effectiveness of CBTp vs TAU, the model required parameters on treatment efficacy (including relapse and recovery), health state utilities and costs. The targeted literature review provided key clinical inputs either directly as transition probabilities between health states or as calibration values (e.g., ratios of prevalence

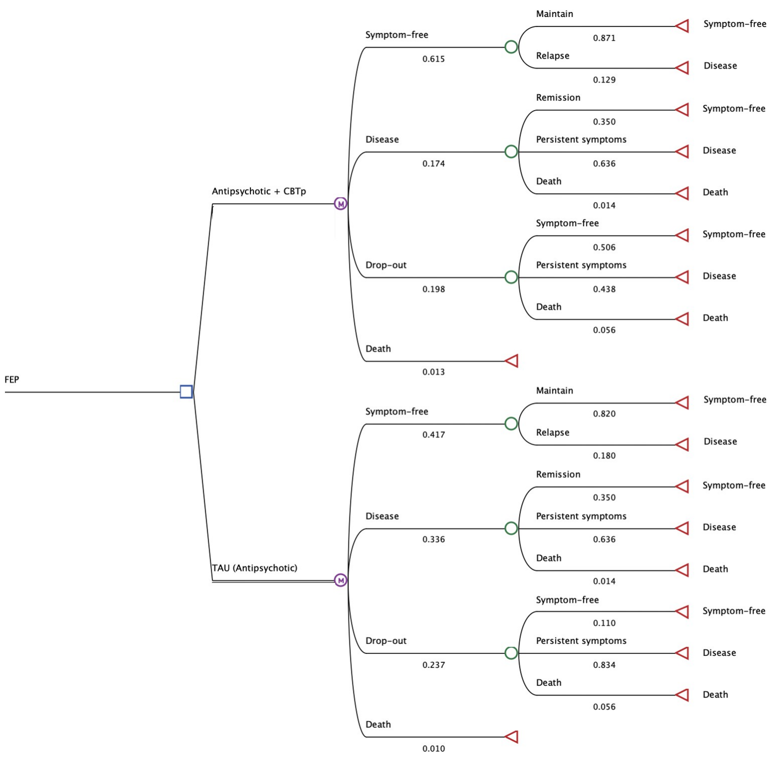


**Figure 1:** *Markov probability tree*. The model begins with first episode psychosis (FEP) patients who receive either CBTp+TAU or TAU. From each treatment arm, patients transition through the labelled health states with subsequent cycles allowing for relapse, remission, persistent symptoms, or death. The blue square is a decision node. Each of its branches represent a strategy which will be evaluated separately to determine the optimal strategy at the decision node. Purple "M" nodes are Markov nodes. Green circles represent chance nodes, where the outcome is unknown but all possibilities are shown as branches. Transition probabilities are shown for each branch. Red triangles denote terminal nodes, which represent the end of a cycle from which a jump state may then return the cohort to a health state for the next cycle.

between states). Evidence regarding CBTp's effect on relapse in FEP is mixed, but several systematic reviews have assessed CBTp efficacy when delivered alongside standard care. The meta-analysis by Mayer et al. (2024)[2] was selected as the principal source for transition probabilities following FEP interventions.

Model inputs from Mayer et al. (2024)[2] were validated against the meta-analysis by Fusar-Poli et al. (2016)[3], which synthesised 82 studies (11,133 patients) with transition rates at 6 months, 1 year and 2–3 years. Fusar-Poli et al. estimated a recurrence risk of 0.42 (95% CI 0.30–0.54) after 1, 2 and 3 years. These estimates were used as a plausibility check rather than directly input, as they did not provide full data for other FEP health states or

stage-specific prevalence. Transition probabilities from Mayer et al. (2024)[2] were used as base-case model parameters. Post drop-out transition probabilities required calibration using relative risks reported by Wijnen et al. (2020)[4] and Robinson et al. (1999)[5]. Annual probabilities obtained from the literature were converted into 3-month cycle probabilities within TreeAge Pro. Complete transition parameters for each treatment pathway are presented in **Table S1.**

**Table S1:** *Transition probabilities for the CBTp plus TAU and TAU groups*

| Transition Parameter | Mean | SD | Distribution | Justification |
|---|---|---|---|---|
| **CBTp plus TAU Group** | | | | |
| FEP to Symptom-free | 0.615 | 0.185 | Beta | Mayer et al. (2024)[2] |
| FEP to Disease | 0.174 | 0.052 | Beta | Mayer et al. (2024)[2] |
| FEP to Drop-out | 0.198 | 0.059 | Beta | Mayer et al. (2024)[2] |
| FEP to Death | 0.013 | 0.004 | Beta | Mayer et al. (2024)[2] |
| Disease to Remission | 0.350 | 0.105 | Beta | Wijnen et al. (2020)[4] |
| Disease to Persistent Symptoms | 0.636 | 0.191 | Beta | Wijnen et al. (2020)[4] |
| Disease to Death | 0.014 | 0.004 | Beta | Wijnen et al. (2020)[4] |
| Drop-out to Symptom-free | 0.506 | 0.152 | Beta | Robinson et al. (1999)[5] |
| Drop-out to Persistent Symptoms | 0.438 | 0.131 | Beta | Robinson et al. (1999)[5] |
| Drop-out to Death | 0.056 | 0.017 | Beta | Robinson et al. (1999)[5] |
| **TAU Group** | | | | |
| FEP to Symptom-free | 0.417 | 0.125 | Beta | Mayer et al. (2024)[2] |
| FEP to Disease | 0.336 | 0.101 | Beta | Mayer et al. (2024)[2] |
| FEP to Drop-out | 0.237 | 0.071 | Beta | Mayer et al. (2024)[2] |
| FEP to Death | 0.010 | 0.003 | Beta | Mayer et al. (2024)[2] |
| Disease to Remission | 0.350 | 0.105 | Beta | Mayer et al. (2024)[2] |
| Disease to Persistent Symptoms | 0.636 | 0.191 | Beta | Mayer et al. (2024)[2] |
| Disease to Death | 0.014 | 0.004 | Beta | Mayer et al. (2024)[2] |
| Drop-out to Symptom-free | 0.110 | 0.033 | Beta | Robinson et al. (1999)[5] |
| Drop-out to Persistent Symptoms | 0.834 | 0.250 | Beta | Robinson et al. (1999)[5] |
| Drop-out to Death | 0.056 | 0.017 | Beta | Robinson et al. (1999)[5] |

*Abbreviations:* FEP, first episode psychosis; TAU, treatment as usual; CBTp, cognitive behavioural therapy for psychosis; SD, standard deviation.

**Table S2:** *Utility parameters used in the model*

| State | Utility Value | SD | Distribution | Source |
|---|---|---|---|---|
| Remission/Symptom-free | 0.756 | 0.227 | Beta | Wijnen et al. (2020)[4] |
| Disease/Persistent Symptoms | 0.362 | 0.109 | Beta | Wijnen et al. (2020)[4] |
| Death | 0 | 0 | Beta | Wijnen et al. (2020)[4] |

*Abbreviations:* SD, standard deviation.

## S4. Utility Parameters

Utilities in this model were derived from psychosis populations using generic preference-based instruments (e.g., EQ-5D). Utility weights were assigned to the three model health states: remission/symptom-free, disease/persistent symptoms and death. The complete health state utilities are presented in Table S2.

## S5. Direct Cost Parameters and Costing Assumptions

Cost parameters were obtained from nationally recognised sources, including the Personal Social Services Research Unit (PSSRU) *Unit Costs of Health and Social Care* (2024 edition)[6], NHS Agenda for Change pay bands[7] and NICE[8]. In the model, direct costs covered all healthcare resources used in delivering CBTp and pharmacotherapy within EIP:

- Consultant psychiatrist reviews
- Routine blood tests and monitoring
- Antipsychotic drug costs
- CBTp delivery by a clinical psychologist

Where possible, a micro-costing approach was applied, with national reference costs used when detailed data were unavailable.

Labour costs were estimated using data on publicly funded clinicians from published aggregates, job postings and expert consultation. In England:

- The average annual salary of a clinical psychologist was approximately £46,148.[9].
- The average annual salary of a consultant general psychiatrist was approximately £123,000.[10]

Clinicians do not spend 100% of their contracted hours on direct patient care. To calculate the direct cost of clinical time, an hourly rate was derived by adjusting for the proportion of time spent in direct work. For example, for a clinical psychologist:

1. A full-time NHS clinical psychologist works 2,080 hours per year (40 hours × 52 weeks).
2. With 60% of time spent on direct work, this equals 1,248 direct clinical hours.
3. With an annual salary of £46,148, the direct hourly cost is:

   £46,148 ÷ 1,248 = £36.98 per direct clinical hour.

In the model, CBTp was assumed to consist of 16 weekly one-hour individual sessions delivered in person by a Band 7 clinical psychologist. The cost of CBTp professional time was therefore calculated as: £36.98 × 16 = £591.68 per patient.

TAU total cost (pharmacotherapy only) combined costs of psychiatrist reviews, drug costs and laboratory monitoring. Drug costs were obtained from the British National Formulary (BNF)[11] and the June 2025 edition of NHS Business Services Authority prescription cost data.[12]

Costs for blood tests and pathology were based on expert opinion and NICE guidelines, assuming twice-yearly comprehensive metabolic panels, including full blood count (FBC), urea and electrolytes (U&Es), lipid profile, fasting glucose, prolactin, thyroid function tests (TFTs) and liver function tests (LFTs) per patient. The full direct cost parameters used in the model are shown in **Table S3.**

**Table S3:** *Detailed direct costs used in the model*

| Parameter | Annual Cost, £ | Distribution | SD | Source | Assumptions |
|---|---|---|---|---|---|
| Olanzapine 20mg/day | 334.60 | Gamma | 100.38 | BNF[11], NHSBSA[12] | One year's supply of drug. |
| Aripiprazole 10mg/day | 231.00 | Gamma | 69.30 | BNF[11], NHSBSA[12] | One year's supply of drug. |
| Risperidone 4mg/day | 147.00 | Gamma | 44.10 | BNF[11], NHSBSA[12] | One year's supply of drug. |
| CBTp | 591.68 | Gamma | 117.50 | PSSRU[6] | Assumes 16 one-hour sessions delivered by a Band 7 clinical psychologist, with a salary of £46,148. |
| Psychiatrist review | 461.26 | Gamma | 138.38 | NHS Health careers[10] | Consultant general psychiatrist with a salary of £123,000 doing half-hour reviews with the patient every two months. |
| Blood tests and monitoring | 101.00 | Gamma | 30.30 | NICE[13] | Assumes two visits made per year for a comprehensive metabolic panel including a FBC, U&Es, lipids, glucose, prolactin, TFT and LFT. |

*Abbreviations:* BNF, British National Formulary; CBTp, Cognitive Behavioural Therapy for psychosis; FBC, Full Blood Count; LFT, Liver Function Tests; NHSBSA, NHS Business Services Authority; PSSRU, Personal Social Services Research Unit; SD, Standard Deviation; TFT, Thyroid Function Tests; U&Es, Urea and Electrolytes.

## S6.Indirect Cost Parameters and Societal Perspective

Estimating indirect costs associated with FEP in EIP was necessary to implement a societal perspective. Indirect costs reflect:

- Lost employment and earnings (absenteeism)
- Reduced productivity of informal carers
- Travel and non-staff costs associated with care
- Overheads and indirect clinical work.

A simplified human capital framework, based on Bonin (2017),[14] was used. Bonin's original framework for anorexia nervosa in England incorporated educational losses and lifetime employment trajectories. Full implementation was not feasible here given limited evidence on presenteeism, educational attainment or long-term welfare dependency in FEP. Therefore, the model prioritised components with the strongest empirical foundations and applied conservative assumptions where evidence was weaker, to avoid adding complexity that would increase uncertainty and obscure key cost drivers. Indirect costs were considered across both the healthcare system and wider society:

- For the healthcare system, indirect work (activities supporting patient care without direct contact) was costed using PSSRU estimates.
- Direct overheads included administration and management, office costs and utilities such as water, gas and electricity.
- Indirect overheads included general management and support services.

The model applied PSSRU overhead values:[6]

- Direct overheads at 29% of direct salary costs.

- Indirect overheads at 16% of direct salary costs.
- Capital overheads of £3,191 representing new-build and land requirements for a local authority office and shared facilities.

Beyond the healthcare system, societal costs included travel to appointments, informal care and lost employment productivity. Absenteeism was the largest component, capturing time away from paid work due to illness, treatment attendance and recovery. Absenteeism costs were based on the median salary for 22–29-year-olds in the UK using Office for National Statistics (ONS) data.[15] Travel costs assumed bus transport to all appointments at a fare of £2.50 per journey.[16]

Informal care, a substantial burden for families of FEP patients, was valued by assigning a monetary cost to unpaid caregiving hours. Using the PSSRU rate of £32.00 per hour, the model assumed carers provided 1.5 hours of informal care per day.

Overall, indirect costs amounted to approximately £17,030 per FEP patient per year. When combined with direct healthcare costs, the total societal cost exceeded £50,000 per FEP patient annually. The full indirect cost parameters used in the model are shown in **Table S4.**

**Table S4:** *Detailed indirect costs used in the model*

| Parameter | Annual Cost, £ | Distribution | SD | Source | Assumptions |
|---|---|---|---|---|---|
| Indirect work (psychologist) | 394.45 | Gamma | 118.34 | PSSRU[6] | 60% of time spent on direct work. |
| Indirect work (psychiatrist review) | 738.02 | Gamma | 221.41 | PSSRU[6] | 1:1.6 ratio of direct to indirect work. |
| Direct overheads (psychologist) | 171.59 | Gamma | 51.48 | PSSRU[6] | Assumed to be 29% of direct salary cost for community-based staff. |
| Direct overheads (psychiatrist review) | 133.77 | Gamma | 40.13 | PSSRU[6] | Assumed to be 29% of direct salary cost for community-based staff. |
| Indirect overheads (psychologist) | 94.67 | Gamma | 28.40 | PSSRU[6] | Assumed to be 16% of direct salary cost for community-based staff. |
| Indirect overheads (psychiatrist review) | 73.80 | Gamma | 22.14 | PSSRU[6] | Assumed to be 16% of direct salary cost for community-based staff. |
| Capital overheads | 3,191.00 | Gamma | 957.30 | PSSRU[6] | Based on the new-build and land requirements for a local authority office and shared facilities for waiting, interviews and clerical support. |
| Non-staff costs | 17,520 | Gamma | 5,256.00 | PSSRU[6] | Based on PSSRU-estimated carer informal carer wages of £32.00 per hour, assumes carer works for 1.5 hours per day. |
| Travel to appointment | 110.00 | Gamma | 33.00 | Regional estimate[16] | Assumes patient travels to appointments by bus, average fare of £2.50. |
| Lost employment | 28,918.00 | Gamma | 8,675.4 | ONS[15] | Median salary for 22-29 year-olds in the UK. |

*Abbreviations:* ONS, office for national statistics; PSSRU, personal social services research unit; SD, standard deviation.

## S7. Economic Evaluation

Cost-effectiveness was expressed using the incremental cost-effectiveness ratio (ICER): the additional cost of an intervention divided by the additional QALYs gained compared to the next least costly option. An intervention is cost saving if it provides greater health benefits at a lower cost. When one option is not clearly superior, cost-effectiveness depends on the decision-maker's willingness-to-pay (WTP) for a QALY. While NICE does not stipulate a maximum WTP, interventions with an ICER below £20,000 per QALY gained are considered cost-effective. For ICERs between £20,000-£30,000, considering additional contextual factors is recommended.[17] These include:

- The quality and robustness of clinical effectiveness evidence, with stronger evidence supporting acceptance of higher ICERs.
- Whether QALYs fully capture the intervention's broader benefits, including social functioning, family relationships, and patient-reported experiences.
- Whether CBTp represents a step-change in care delivery that provides broader system benefits not captured in the model.
- Equity considerations regarding whether CBTp addresses unmet clinical need or provides particular benefit to disadvantaged population groups.

The findings of this cost-effectiveness analysis (CEA) are generalisable to adults with FEP in the UK who are under the care of EIP and can both receive antipsychotic medication and engage in CBTp.

## S8. Deterministic Sensitivity Analysis

One-way deterministic sensitivity analysis assessed how individual parameters influenced cost-effectiveness. Each parameter was varied across its plausible range while other parameters were kept at base values, using a 30% standard deviation to define ranges.

Results were presented as tornado diagrams, ranking parameters by their ICER impact. Threshold analyses were performed to identify values where cost-effectiveness conclusions changed, particularly the minimum clinical effectiveness required for CBTp to achieve cost-effectiveness at different WTP thresholds.

## S9. Probabilistic Sensitivity Analysis – Technical Details

Parameter uncertainty was handled by defining assigning a probability distribution to all model inputs for the probabilistic sensitivity analysis (PSA). Beta distributions were used for utility values. Transition probabilities were modelled using a multivariate (Dirichlet) beta distributions. Costs were assigned gamma distributions to allow only non-negative values whilst maintaining the right-skewed characteristics typical of cost data.

Monte Carlo simulation was then used for the PSA. Markov chain Monte Carlo (MCMC) repeatedly samples from probability distributions to model uncertain outcomes. In this analysis, it allowed simulations to be propagated across multiple correlated parameters while maintaining the interdependencies between transition probabilities, costs, and utilities. Each parameter was treated as a random variable with its own distribution, and repeated sampling generated thousands of plausible parameters sets. The model ran over 100,000 Monte Carlo iterations, producing 100,000 ICERs.

The PSA results could be visually represented using two key plots. The cost-effectiveness acceptability curve (CEAC), used to present ICER uncertainty, displayed the proportion of iterations in which the CBTp intervention was cost-effective across a range of WTP thresholds. A cost-effectiveness plane was also generated, plotting incremental costs against incremental QALYs for individual simulations, providing an overview of ICER uncertainty and the joint distribution of costs and effects.

## References

[1] Alvarez-Jimenez M, Priede A, Hetrick SE, Bendall S, Killackey E, Parker AG, et al. “Risk Factors for Relapse Following Treatment for First Episode Psychosis: A Systematic Review and Meta-analysis of Longitudinal Studies”. In: *Schizophrenia Research* 139.1–3 (2012), pp. 116–128. DOI: 10.1016/j.schres.2012.05.007. https://doi.org/10.1016/j.schres.2012.05.007.

[2] Mayer SF, Corcoran C, Kennedy L, Leucht S, and Bighelli I. “Cognitive behavioural therapy added to standard care for first-episode and recent-onset psychosis”. In: *Cochrane Database of Systematic Reviews* 3 (2024). DOI: 10.1002/14651858.CD015331.pub2. https://doi.org/10.1002/14651858.CD015331.pub2.

[3] Fusar-Poli P, Cappucciati M, Bonoldi I, Hui LM, Rutigliano G, Stahl DR, et al. “Prognosis of Brief Psychotic Episodes: A Meta-analysis”. In: *JAMA Psychiatry* 73.3 (2016), pp. 211–220. DOI: 10.1001/jamapsychiatry.2015.2313.

[4] Wijnen BFM, Thielen FW, Konings S, Feenstra T, Van Der Gaag M, Veling W, et al. “Designing and Testing of a Health-Economic Markov Model for Prevention and Treatment of Early Psychosis”. In: *Expert Review of Pharmacoeconomics & Outcomes Research* 20.3 (2020), pp. 269–279. DOI: 10.1080/14737167.2019.1632194. URL: https://doi.org/10.1080/14737167.2019.1632194.

[5] Robinson D, Woerner MG, Alvir JM, Bilder R, Goldman R, Geisler S, et al. “Predictors of relapse following response from a first episode of schizophrenia or schizoaffective disorder”. In: *Archives of General Psychiatry* 56.3 (1999), pp. 241–247. DOI: 10.1001/ archpsyc.56.3.241.

[6] Dargan A. *Unit Costs of Health and Social Care 2023 Manual.* Report. Personal

Social Services Research Unit (PSSRU), University of Kent, 2024. DOI: 10.22024/UniKent/ 01.02.105685. https://kar.kent.ac.uk/id/eprint/105685.

[7] NHS Health Careers. *Agenda for Change - pay rates*. https://www.healthcareers. nhs.uk/working-health/working-nhs/nhs-pay-and-benefits/agenda-change- pay-rates. Accessed: 19 June 2025. 2025.

[8] National Institute for Health and Care Excellence (NICE). *Psychosis and schizophrenia in adults: prevention and management*. NICE Clinical Guidelines, No. 178. London:National Institute for Health and Care Excellence (NICE), Mar. 2014. https://www.ncbi.nlm.nih.gov/books/NBK555203/.

[9] Prospects.ac.uk. *Job Profile: Clinical Psychologist*. https://www.prospects.ac.uk/ job-profiles/clinical-psychologist. Accessed: 27 May 2025. 2025.

[10] NHS Health Careers. *Pay for doctors*. https : / / www . healthcareers . nhs . uk / explore-roles/doctors/pay-doctors. Accessed: 30 May 2025. 2025. https: //www.healthcareers.nhs.uk/explore-roles/doctors/pay-doctors.

[11] Joint Formulary Committee. *British national formulary (BNF 89) March 2025*. en. London, England: Pharmaceutical Press, Mar. 2025.

[12] NHS Business Services Authority. *NHS Electronic Drug Tariff*. Accessed: 2025-06-12. https : / / www . nhsbsa . nhs . uk / sites / default / files / 2025 - 06 / Drug % 20Tariff%20July%202025.pdf.

[13] National Institute for Health and Care Excellence (NICE). *NG45: Routine Preoperative Tests for Elective Surgery – Guideline Appendices 13*. Appendix 13 to NICE Guideline NG45. Preoperative tests, Economic considerations. London, UK: National Institute for Health and Care Excellence, Apr. 2016. https://www.nice.org.uk/guidance/ ng45/documents/guideline-appendices-13.

[14] Bonin E.-M. “The societal costs of Anorexia nervosa in England”. Available at https:

//etheses.lse.ac.uk/3766/1/Bonin___the-societal-costs-of-anorexia.pdf. PhD thesis. London, England: London School of Economics and Political Science, 2017.

[15] Office for National Statistics. *Earnings and hours worked, age group: ASHE Table 6*. https://www.ons.gov.uk/employmentandlabourmarket/peopleinwork/earningsandworkinghou datasets/agegroupashetable6. Released 29 October 2024; accessed 2025-07-01. 2024.

[16] Stagecoach East. *Never pay more than £3 for your single journey*. https:// www . stagecoachbus.com/promos-and-offers/east/never-pay-more-than-3-pounds- for-your-single-journey. Accessed: 2025-06-21. 2025.

[17] National Institute for Health and Care Excellence. *NICE Health Technology Evalu- ations: The Manual*. Updated October 31, 2023; accessed [April 14, 2025]. National Institute for Health and Care Excellence. London, England, Jan. 2022.
